# Rapid inactivation of aerosolised influenza virus using low-concentration gaseous hypochlorous acid

**DOI:** 10.1038/s41598-025-19020-8

**Published:** 2025-09-29

**Authors:** Koki Narihata, Masaru Minamiguchi, Miki Hata, Mitsuhiko Ueda, Shinji Yoshida, Yoshihiro Sakoda

**Affiliations:** 1 R&D Center, Indoor Air Quality Business Division, Panasonic Ecology Systems Co., Ltd., Kasugai, Aichi 486-8522 Japan; 2 Green Transformation Division, Panasonic Holdings Corporation, Moriguchi, Osaka 570-8501 Japan; 3 Laboratory of Microbiology, Department of Disease Control, Faculty of Veterinary Medicine, Hokkaido University, Sapporo, 060-0818 Japan; 4 One Health Research Center, Hokkaido University, Sapporo, 060-0818 Japan; 5 International Collaboration Unit, International Institute for Zoonosis Control, Hokkaido University, Sapporo, 001-0020 Japan; 6 Hokkaido University Institute for Vaccine Research and Development (HU-IVReD), Hokkaido University, Sapporo, 001-0021 Japan

**Keywords:** Influenza virus, Influenza virus, Isolation, separation and purification, Microbiology techniques

## Abstract

Effective infection control measures are crucial for limiting pathogen transmission, including aerosol infections. Several instances of the use of hypochlorous acid solution spray to inactivate airborne viruses have been reported. However, its effectiveness in controlling infections in aerosol transmission scenarios remains unclear. We evaluated the efficacy of gaseous hypochlorous acid [HOCl_(g)_], which is safe for occupied environments, in infection control. Exposure to 10–20 ppb HOCl_(g)_ for several seconds significantly reduced the infectivity of aerosolised H1N1 influenza A virus in water-rich droplets by 2.09–2.79 logs. However, no significant reduction occurred in dry aerosols after water evaporation. A stronger inactivation effect was noted at 50% compared to 30% relative humidity, with a 3.06-fold increase in effectiveness. Comparative analysis with a reactive oxygen species gas (O₃), possessing lower liquid-phase solubility than HOCl_(g)_, suggested that aerosol water content facilitates HOCl-mediated virucidal activity. Viral infectivity decreased by 2.34 logs under 20-ppb conditions, even in the presence of 0.3% mucin. These findings underscore the effectiveness of HOCl_(g)_ against aerosolised H1N1 influenza A virus and its potential for infection control.

**Supplementary Information:**

The online version contains supplementary material available at 10.1038/s41598-025-19020-8.

## Introduction

Severe acute respiratory syndrome coronavirus 2 (SARS-CoV-2) is responsible for the global COVID-19 pandemic^1^. This crisis has heightened awareness of infection transmission through “infectious respiratory particles”—droplets and aerosols generated by coughing, sneezing, and speaking^2^. Aerosols containing SARS-CoV-2 can disperse over a wide area via air currents and remain infectious for several hours^3^. A previous study detected viable SARS-CoV-2 in the air surrounding patients in the early stages of infection^4^. The H1N1 influenza A virus, which is also an enveloped virus, caused the pandemic in 2009. As reports suggest that half of the transmission events caused by influenza A virus in households occur through aerosol transmission, influenza A virus primarily spread through infectious respiratory particles^5–7^. Strategies such as social distancing, ventilation, and mask use have been implemented to mitigate transmission. However, a more effective approach is needed—one that inactivates viral aerosols at the source before they reach new hosts. This method must be suitable for occupied spaces and capable of covering large areas, given the unpredictability of viral particle release into the air by human hosts. Considering these factors, we focused on gaseous hypochlorous acid (HOCl_(g)_) as a potential solution.

Physical treatment with UV light and plasma-generated reactive oxygen species (ROS) as chemical disinfectants exhibit broad inactivation effects. However, their applicability in human environments is limited because of the health risks associated with human exposure and potential damage to various material surfaces^8,9^. 

HOCl, even in its liquid form, exhibits low irritation and toxicity to humans and reportedly possesses broad-spectrum antimicrobial activity against fungi, spores, and viruses^10–14^. HOCl has also been shown to react with proteins, lipid envelopes, and nucleic acids, leading to the inactivation of influenza viruses, coronaviruses, and adenoviruses^15–18^.

Hypochlorous acid solution (HOCl_(aq)_) volatilises at 30 °C, with a vapour pressure of 1.25–12.52 Pa when its concentration ranges from 0.011 to 0.11 mol/L^19^. The resulting HOCl_(g)_ disperses into the air without causing moisture accumulation, thereby allowing it to act over a wide area and reportedly also being capable of inactivating airborne viruses^20,21^. The safety standard for HOCl_(g)_ follows that of chlorine gas. According to the European Union Risk Assessment Report on chlorine, 0.5 ppm is considered a non-hazardous level^20^. Additionally, the *Guidelines for space purification using hypochlorite water (electrolyzed water)*, 1st Edition (Safety Information Edition), compiled by the Functional Water Foundation, a Japanese organisation involved in spreading awareness of HOCl technologies, set the upper limit for long-term continuous exposure (over 14 days) to volatilised HOCl at 50 ppb for chlorine gas concentration^22^.

The disinfecting efficacy of HOCl_(g)_ is influenced by humidity, with higher humidity levels enhancing its effectiveness^23^. Yoshida et al*.*^24^ suggested that increased humidity increases the moisture content on target surfaces, facilitating the dissolution and concentration of HOCl_(g)_, thereby enhancing its disinfecting action. On this basis, we hypothesised that HOCl_(g)_ would be highly effective in inactivating aerosolised viruses with high water content immediately after their release as droplets, as it would dissolve into the droplets. Thus, for this mechanism to be effective, the active substance must dissolve in water-rich aerosols.

Several studies have reported examples of using hypochlorous acid solution spray to inactivate airborne viruses^25,26^. However, these studies include the effect of hypochlorous acid mist, required at least several minutes for inactivation, or were conducted under conditions where it was unclear whether the concentrations were suitable for use in occupied environments. Consequently, questions regarding their effectiveness in controlling infections in aerosol transmission scenarios persist.

The purpose of this study was to identify conditions under which HOCl_(g)_, at concentrations suitable for use in occupied environments, can achieve inactivation within a matter of seconds, thereby providing an option as a new infection control measure. Additionally, we aim to provide information that contributes to elucidating the principles behind these inactivation conditions by being the first report to clearly separates the presence or absence of moisture contained in aerosol.

In this study, we investigated the inactivation effect of volatilised HOCl_(g)_ on aerosols containing H1N1 influenza A virus generated as water-containing droplets. To assess the impact of water content on effectiveness, we conducted a comparative test using dry aerosols from which water had been removed. Additionally, to evaluate the effect of solubility differences, we performed a comparative study using various ROS, including ozone, generated through corona discharge, as these species are known for their antimicrobial properties^27–29^.

As the effectiveness of HOCl is expected to decrease when aerosols are emitted from humans due to the presence of proteins, we also examined its inactivation effect on aerosols containing 0.3% mucin^25,30^.

## Results and discussion

### Rapid inactivation of influenza A virus at low humidity and low HOCl concentration

**Fig. 1:**
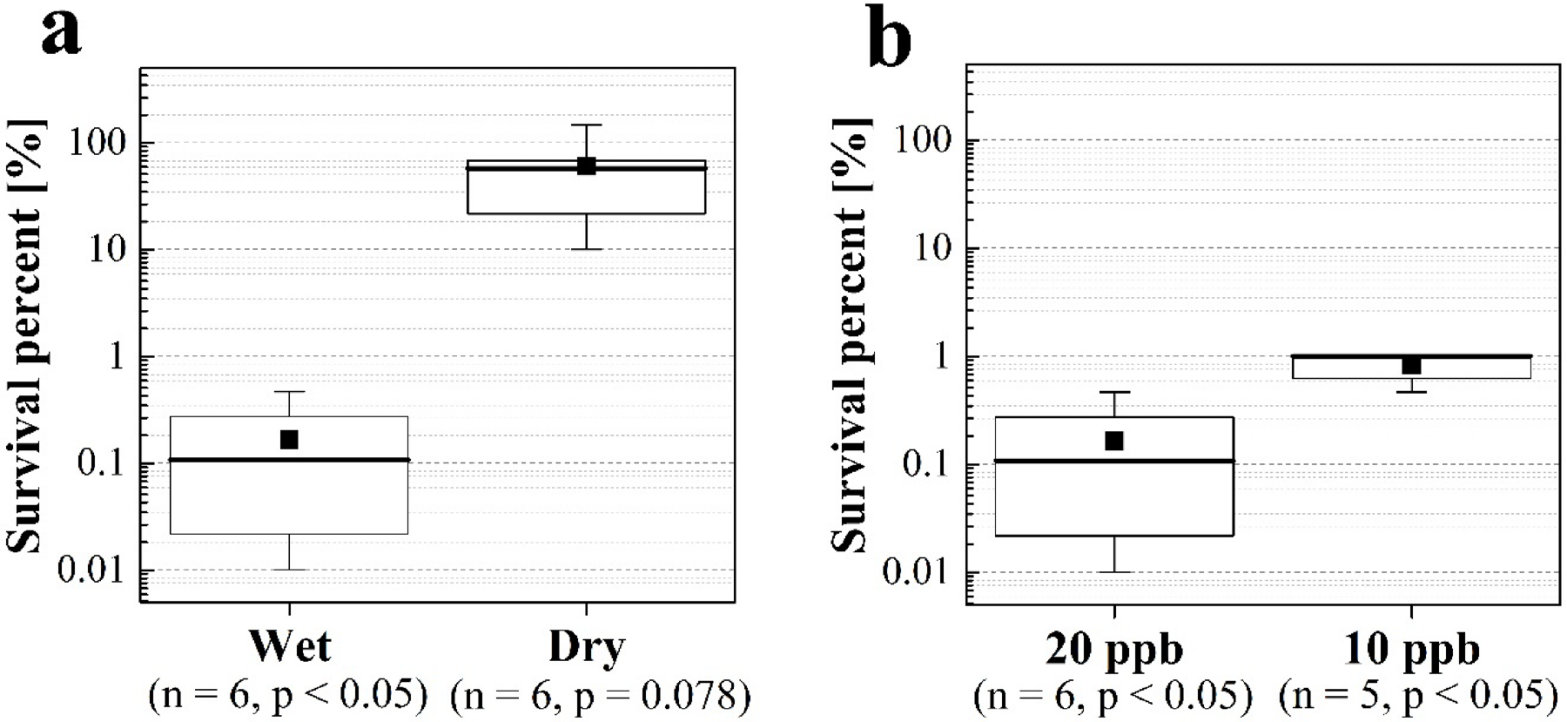
Survival percent of influenza virus (**a**) in wet/dry aerosol subjected to HOCl [20 ppb, 50% relative humidity, 1.8 s] and (**b**) in wet aerosol subjected to 10 and 20 ppb concentrations of HOCl [50% relative humidity, 1.8 s]. The box represents the 25th to 75th percentile range, the whisker plot shows the maximum and minimum values, the thick line within the box denotes the median, and the ■ symbol represents the average value.

We evaluated the survival percent (SP) of H1N1 influenza A virus in moist aerosols and aerosols dried using a diffusion dryer under environmental conditions subjected to a HOCl_(g)_ concentration of 20 ppb at a relative humidity of 50% for a gas contact time of 1.8 s (Fig. 1a). For influenza viruses in moist aerosols, a significant reduction in infectivity of 99.84% was observed compared to that for the 0 ppb blank (Wilcoxon signed-rank test, *p* = 0.016, n = 6, Z = 2.097, r = 0.856). However, no significant reduction in infectivity was detected for viruses in dry aerosols (Wilcoxon signed-rank test, *p* = 0.078, n = 6, Z = 1.468, r = 0.599). The 20-ppb concentration is fully applicable in real human environments. These findings suggest that HOCl_(g)_ effectively inactivates influenza viruses in moist aerosols released by infected individuals. Additionally, the substantial difference between moist and dry aerosols under the same environmental conditions is noteworthy as this implies that the presence of moisture on the virus surface is critical. Wang et al*.*^31^ indicated that the inactivation effect of ClO₂ is linked to water vapour condensing on the surface, acting as a carrier for ClO₂, which is easily dissolved. A similar inactivation process is believed to have contributed to the observed effect of HOCl in the present study. Miyaoka et al*.* also demonstrated that spraying 100–500 ppm HOCl_(aq)_ on aerosolised infectious bronchitis virus containing 0.5% foetal bovine serum (FBS) led to inactivation within a few seconds^25^. This effect can be ascribed to either coagulation between the droplet-shaped airborne virus and microdroplets containing HOCl_(aq)_ prior to volatilisation or to dissolution of HOCl_(g)_ into the virus-laden droplets, both of which reduced infectivity. These findings suggest that the combined effect of the virus, moisture, and HOCl was responsible. In contrast, in the present study, the droplet-form airborne virus came into contact with HOCl_(g)_, which dissolved into the droplets; thus, the combination of virus, moisture, and HOCl contributed to rapid inactivation.

Furthermore, Imoto et al*.* confirmed a 2.56 log inactivation effect against the influenza virus by spraying HOCl_(aq)_ with a contact time of 5 min under low concentration conditions (HOCl_(g)_ : 20ppb)^26^. In contrast to this study, they sprayed microdroplets containing HOCl_(aq)_ onto aerosolised viruses that had evaporated several minutes after spraying. However, both conditions commonly involve the presence of viruses, moisture, and HOCl, suggesting that significant effects can also be expected in low-concentration HOCl gas due to the presence of moisture. Next, we consider real-world spaces and discuss the results for safer and more comfortable gas concentrations, as well as realistic humidity ranges. The SP of H1N1 influenza A virus in aerosols containing moisture under environmental conditions of a HOCl_(g)_ concentration of 10 ppb, gas contact time of 1.8 s, and relative humidity of 50% was evaluated (Fig. 1b). Even with a reduced HOCl concentration of 10 ppb, a significant inactivation effect of over 99% was observed (Wilcoxon signed-rank test, *p* = 0.031, n = 5, Z = 1.923, r = 0.860). Compared to 20 ppb, the inactivation effect was reduced by a factor of 1.33 in terms of logarithmic reduction. This decrease is likely due to the reduction in the equilibrium concentration within the droplet moisture as the air concentration decreases, leading to a lower adsorption rate. The 10-ppb concentration is not only 1/50 of the safety standard concentration but also a level that is detectable by the human sense of smell with minimal discomfort, making it a more realistic concentration for practical use.

**Fig. 2:**
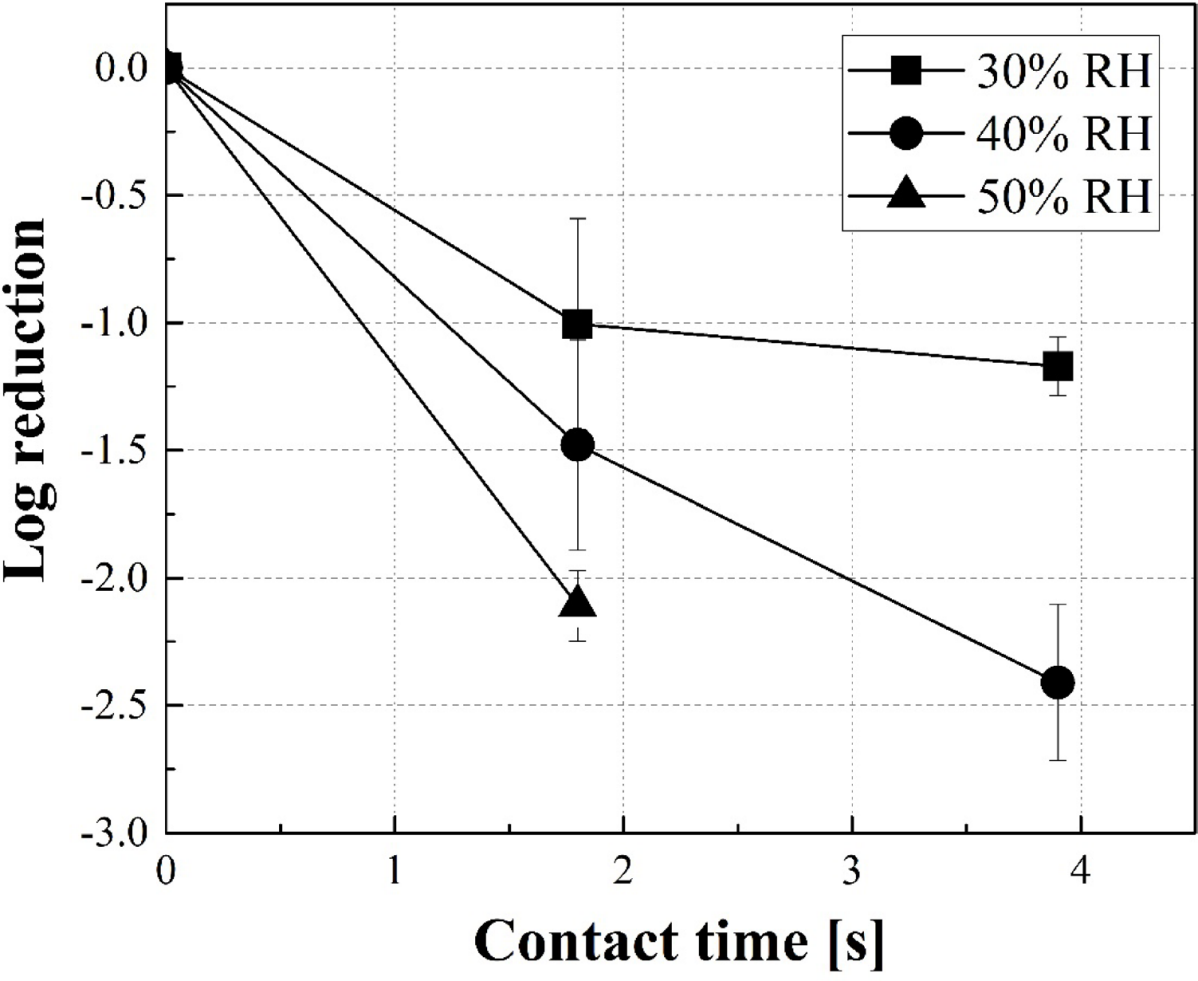
Survival percent of influenza virus in wet aerosol subjected to a HOCl concentration of 10 ppb at different relative humidities (RH) (30, 40, 50%RH) and gas contact times (1.8, 3.9 s). The number of trials (n) was n = 3 for 1.8 s and n = 5 for 3.9 s at 30% RH; n = 8 for 1.8 s and n = 5 for 3.9 s at 40% RH. The data are presented as the mean ± 1 SD of ‘n’ independent samples.

The Log reduction for H1N1 influenza A virus in aerosols containing moisture under environmental conditions of a HOCl_(g)_ concentration of 10 ppb, gas contact times of 1.8 and 3.9 s, and relative humidity ranging from 30 to 50% were evaluated (Fig. 2). The relative humidity range of 30–50% was selected based on the average relative humidity in homes in Japan during winter^32^. Under identical relative humidity conditions, the reaction time was directly proportional to the inactivation effect. Additionally, under the same reaction time, a higher relative humidity resulted in a greater inactivation effect. This can be explained using the concept of CT value, where extending the reaction time increases the contact time between the virus and HOCl, leading to a higher inactivation effect.

Conversely, for droplet-like aerosols containing moisture, the effect of relative humidity was expected to be minimal because the droplets inherently retained the moisture acting as a carrier, and the difference in evaporation time was small—only a few tens of milliseconds. However, a sizeable difference of 3.06-fold was observed between 30 and 50% relative humidity^33^.

Previous studies have shown that the final equilibrium diameter and surface area of aerosol droplets change with evaporation under varying relative humidity conditions, and the degree of inorganic salt crystallisation within the droplets also differs^34,35^. Based on these droplet properties, it is thought that in a higher-humidity environment, the droplets maintain their liquid state. Consequently, this allows more HOCl to readily partition into the aqueous phase, which is considered a major factor in enhancing the inactivation effect. In contrast, in a low-humidity environment (e.g., 30% relative humidity), salts crystallise, thereby promoting the evaporation of moisture from the aerosol. This prevented HOCl_(g)_ from dissolving effectively, leading to a reduced inactivation effect. Furthermore, an increased surface area is believed to contribute to a higher inactivation effect by enhancing the initial adsorption, even in situations where diffusion within the droplet is the rate-limiting step. Additionally, the humidity response of O_3_ gas absorption characteristics, as reported by Shiraiwa et al*.*^36^, suggests that the viscosity of the phase decreases with increasing relative humidity, improving the diffusibility of the components. The partitioning of HOCl from the gas phase is expected to undergo significant changes during the evaporation process. Therefore, future studies should investigate the changes in HOCl concentration and distribution inside the liquid, along with the inactivation mechanism.

Here, we confirmed an inactivation effect of over 99% in just 3.9 s, even in a 40% relative humidity environment, which is considered the lower limit of comfortable humidity according to the American Society of Heating, Refrigeration, and Air-Conditioning Engineers (ASHRAE). The results demonstrate the effectiveness of the inactivation method in a comfortable temperature and humidity environment. In real-world environments, humidification is often used to maintain a comfortable humidity level. As HOCl_(aq)_ is vaporised to supply HOCl to the air, this inactivation method is highly compatible with such humidification practices.

### Comparison with ROS gas to verify solubility differences

Next, we examined whether the difference in solubility of the active substances plays a crucial role in the inactivation effect on these influenza viral aerosols containing significant amounts of water. For comparison with HOCl_(g)_, we used ROS gas (primarily O_3_). The Henry constant of HOCl_(g)_ is much smaller than that of O_3_, thereby making it readily soluble in water^37,38^. The gas–liquid equilibrium concentration at 10 ppb of HOCl_(g)_ was estimated to be 3.3 ppm based on actual measurements^39^. In contrast, at 10 ppb of O_3(g)_, the concentration is below 1 ppb at all pH levels and temperatures, based on the Roth–Sullivan equation. This suggests that the differences in solubility could be adequately compared.

**Fig. 3:**
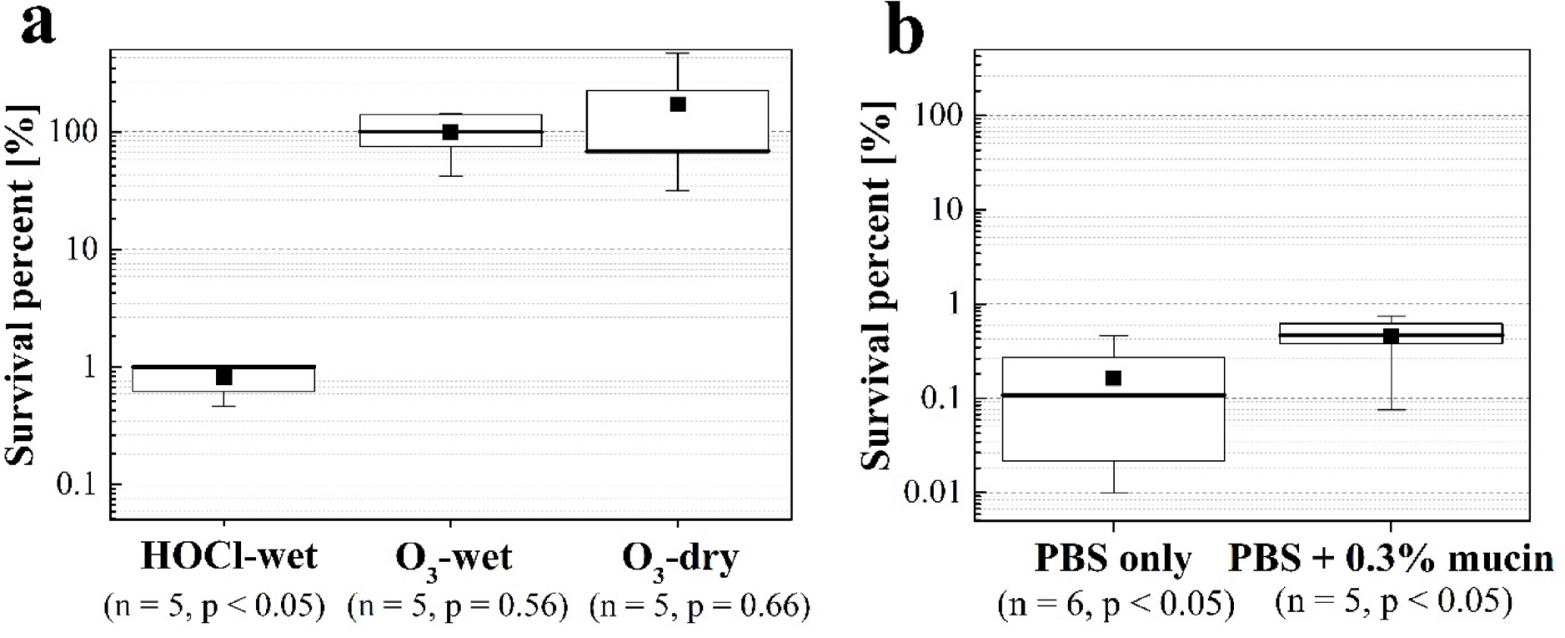
Survival percent of influenza virus (**a**) subjected to different chemical disinfectants [10 ppb, 50% relative humidity, 1.8 s]. (**b**) Subjected to HOCl when 0.3% mucin was added [20 ppb, 50% relative humidity, 1.8 s]. PBS, Phosphate-buffered saline. The box represents the 25th to 75th percentile range, the whisker plot shows the maximum and minimum values, the thick line within the box denotes the median, and the ■ symbol represents the average value.

The inactivation effects of HOCl_(g)_ and ROS gas (O_3_ concentration: 10 ppb) generated by corona discharge against H1N1 influenza A virus in aerosols containing moisture were evaluated (Fig. 3a). We also compared the results for viruses in aerosols dried using a diffusion dryer. The conditions were a gas contact time of 1.8 s and a relative humidity of 50%. In the case of ROS gas, no significant reduction in virus infectivity was observed in either the water-containing (Wilcoxon signed-rank test, *p* = 0.563, n = 5, Z = 0, r = 0) or dry aerosols (Wilcoxon signed-rank test, *p* = 0.656, n = 5, Z = − 0.271, r = − 0.121). In dry aerosols, the lack of water likely explains why no effect was observed with ROS. Based on the CT values reported in previous studies, achieving 99% virus inactivation in dry aerosols with 10 ppb O_3_ is estimated to require 42 min of contact, even in a high-humidity environment, which is consistent with the lack of effect observed in our short-term experiment^29,40^. Conversely, in wet aerosol, the low solubility of O_3_ gas—which accounts for a majority of ROS—may have prevented it from achieving the same rapid inactivation effect as HOCl_(g)_. Based on these results, it is suggested that the high solubility of HOCl_(g)_ is a key factor enabling the inactivation of H1N1 influenza A viruses in wet aerosols during short-term spatial exposure.

Klug et al*.*^41^ evaluated the influence of humidity on O₃ and ClO₂ against MS2 in aerosols supplied at a constant humidity. ClO₂ showed a higher inactivation rate than O₃, even under conditions of lower humidity and concentration than O₃. As ClO₂ is a substance that readily partitions into the aqueous phase, similar to HOCl, it possibly exhibits a higher degradation rate than O₃ under higher humidity conditions, which is consistent with the results of this study.

However, factors other than solubility are also thought to influence the effect, as HOCl and O₃ also differ in chemical properties such as oxidation potential and reaction rate constants. It has been reported that the inactivation by O_3_ under high-humidity conditions may be assisted by hydrolysis, suggesting that its effective environment could differ from the conditions in our experiment^42,43^. Furthermore, Ratnesar-Shumate et al*.*^44^ reported that differences in the ease of water uptake between Bacillus spores and MS2, as well as the hygroscopicity of components contained in the aerosols, might explain differences in the speed of the oxidation process^44^. Therefore, further investigation is necessary, as the conditions under which inactivation is effective may differ not only with the type of gas but also with the physical properties of the target aerosol.

### Effect of the presence of mucin

Thus far, our results have shown that HOCl_(g)_ exhibits a strong inactivation effect on droplet-containing airborne viruses. However, droplets from actual infected individuals contain proteins that are believed to consume HOCl_(g)_. Makimura et al*.*^45^ reported that 0.2% mucin reacts with HOCl_(aq)_, thereby reducing the free chlorine concentration to 1/2600 or less within 1 min^45^. Therefore, we investigated whether HOCl_(g)_ is similarly consumed in an aerosol state and whether its inactivation effect on viruses is inhibited.

We assessed the inactivation effect of HOCl_(g)_ on H1N1 using a nebuliser containing 0.3% mucin to simulate saliva. The inactivation effects of HOCl_(g)_ with and without 0.3% mucin under conditions of 20 ppb, 50% relative humidity, and 1.8 s were evaluated (Fig. 3b). The reduction in inactivation due to the presence of 0.3% mucin was only a 0.45 log reduction compared to that using phosphate-buffered saline (PBS) alone, which confirmed a significant inactivation effect (Wilcoxon signed-rank test, *p* = 0.031, n = 5, Z = 1.888, r = 0.844). Pan et al*.*^30^ reported that when PBS-mucin droplets containing viruses evaporate, a coffee-ring effect is formed, with viruses and mucin distributed on the droplet surface via capillary flow. Yang & Marr^34^ also noted that enveloped viruses tend to accumulate on the droplet surface. During evaporation, the presence of the virus on the droplet surface, which retains moisture, is believed to bring the virus closer to the HOCl_(g)_ adsorbed on the droplet surface and enhanced its reactivity. Despite this, the observed 0.45 log reduction is likely due to mucin consumption of some HOCl_(g)_. Continued evaporation leads to the loss of moisture causing HOCl_(g)_ to no longer dissolve, thereby significantly reducing the inactivation effect.

## Conclusions

We confirmed that volatilised HOCl_(g)_ can inactivate H1N1 influenza A virus in aerosols containing water within a few seconds. Comparisons with dry-state aerosols, various humidity and reaction time conditions, and ROS gases suggest that this effect is unique to HOCl_(g)_, which has high solubility in droplets. Furthermore, we adopted a HOCl_(g)_ concentration of 10 ppb, which is sufficiently low relative to the upper limit of continuous exposure and unlikely to cause discomfort to people. These results were obtained at 40% relative humidity or more, which indicated that the inactivation effect can be achieved safely in environments that people can comfortably occupy. Even when 0.3% mucin was added to simulate saliva, a high inactivation effect was confirmed within a few seconds in a wet state. These results suggest that HOCl_(g)_ is an effective measure for instantaneously inactivating viruses released from infected individuals, thereby reducing the spread of infection.

However, further verification is required to confirm the feasibility of this method as an effective countermeasure. First, although airflow was present, the verification was conducted in a closed duct space. The diffusion and adsorption processes of aerosols and HOCl_(g)_ are believed to differ in larger spaces; therefore, testing in a more expansive environment is necessary. Second, the effects of components other than HOCl_(g)_ should not be ruled out, and additional evaluations using different methods to generate HOCl_(g)_ are required. Third, although saliva was simulated using a simplified PBS + mucin solution, its actual effect on droplets could not be fully reproduced experimentally. The interactions between various components, the viruses within, and the aerosol properties of droplets generated during evaporation are more complex. Fourth, only the A/Puerto Rico/1968 (H1N1) was tested in the present study, and it is desirable to demonstrate the efficacy of HOCl_(g)_ against other viral strains and non-enveloped viruses. Fifth, the effects of temperature have not been evaluated. It has been reported that hypochlorous acid exhibits increased reactivity at higher temperatures^23^. However, the solubility and evaporation characteristics in droplets are also thought to be influenced by temperature, indicating the need for further evaluation. Furthermore, it is essential to evaluate the effectiveness of infection suppression using animal models in future studies^46^.

Nonetheless, to the best of our knowledge, this study represents the first report of a rapid (i.e., within seconds) inactivation effect in an environment with a gas concentration and humidity feasible in real spaces. These findings are expected to improve our understanding of the inactivation process by HOCl_(g)_ and contribute to the development of effective countermeasures.

Although this study focused on only one strain of influenza virus, previous research has shown that HOCl reacts with cell membranes and nucleic acids and is effective against various microorganisms. Specifically, SARS-CoV-2 shares several similarities with influenza viruses, including lipid bilayers, structural proteins, and nucleoproteins. Consequently, HOCl is expected to be just as effective in inactivating SARS-CoV-2.

## Methods

### Preparation of disinfectants

HOCl_(aq)_ was prepared by adjusting the pH of a NaOCl solution (FUJIFILM Wako Pure Chemical Corporation, Osaka, Japan) to 5.5–6.0 using 1 M hydrochloric acid (FUJIFILM Wako Pure Chemical Corporation), followed by dilution with ultrapure water to approximately 100 ppm. Subsequently, HOCl_(g)_ was generated through volatilisation by bubbling clean air into the adjusted HOCl_(aq)_.

ROS were produced via corona discharge. A high voltage of 4.5–5.0 kV was applied to the discharge electrode using a custom-built corona discharge device equipped with a discharge electrode and ground electrode. The discharge current ranged from 12.2 to 16.7 µA.

### Virus

The A/Puerto Rico/1968 (H1N1) strain of influenza A virus was selected as the test virus. The virus was inoculated into the allantoic cavity of 9-day-old chicken eggs (Iwamura Hatchery co., ltd., Hokkaido, Japan) and incubated at 35 °C for 48 h. The hens producing embryonated eggs were raised under the conventional conditions without vaccination against avian influenza. The propagated virus was clarified by centrifugation, aliquoted, and stored at − 80 °C until use. The infectious virus concentration was quantified using Madin–Darby canine kidney (MDCK) cells, and the viral infection titre was measured as median tissue culture infectious dose (TCID_50_). At the time of testing, the frozen stock was thawed and diluted in 1 × PBS or that containing 0.3% mucin (FUJIFILM Wako Pure Chemical Corporation) for testing. The viruses used for spraying were adjusted to a concentration of 10^6.7^–10^7.5^ TCID_50_/mL, with a volume of 40 mL. When diluted in 1 × PBS, the total protein concentration was approximately 1 µg/mL, as measured using a Bradford assay.

MDCK cells were cultured in Eagle’s minimum essential medium (EMEM) (Shimadzu Diagnostics, Kyoto, Japan) supplemented with 10% FBS (Sigma-Aldrich, St. Louis, MO, USA), 1.85 mM L-glutamine (Nacalai Tesque, Kyoto, Japan), 90 units/mL penicillin and 90 µg/mL streptomycin (Meiji Seika Pharma, Tokyo, Japan), 7.20 µg/mL gentamicin (Takata Pharmaceutical, Saitama, Japan), and 1.25 g/L sodium bicarbonate (FUJIFILM Wako Pure Chemical Corporation) at 37 °C and 5% CO_2_ in a CO_2_ incubator. MDCK cells cultured in 96-well plates for 96 h were washed with 1 × PBS and then titrated with 100 µL of a tenfold serial dilution solution of the virus sample. Dilutions were made using the EMEM medium described above without 10% FBS and supplemented with 1.25 µg/mL acetyl trypsin (Sigma-Aldrich). For virus samples that were not diluted in the medium, the solution was removed 1 h after titration, and 100 µL of the medium used for dilution was added. After 72 h of incubation, the cytopathic effect (CPE) of MDCK cells was measured using an inverted microscope (ECLIPSE Ts2; Nikon Solutions Co., Ltd., Tokyo, Japan). The TCID_50_ was calculated based on the observed CPE using the Reed–Muench method. All infectious virus experiments were performed in a biosafety cabinet at a Biosafety Level 2 (BSL-2) facility.

### Evaluation of airborne virus inactivation

#### Environmental control

**Fig. 4:**
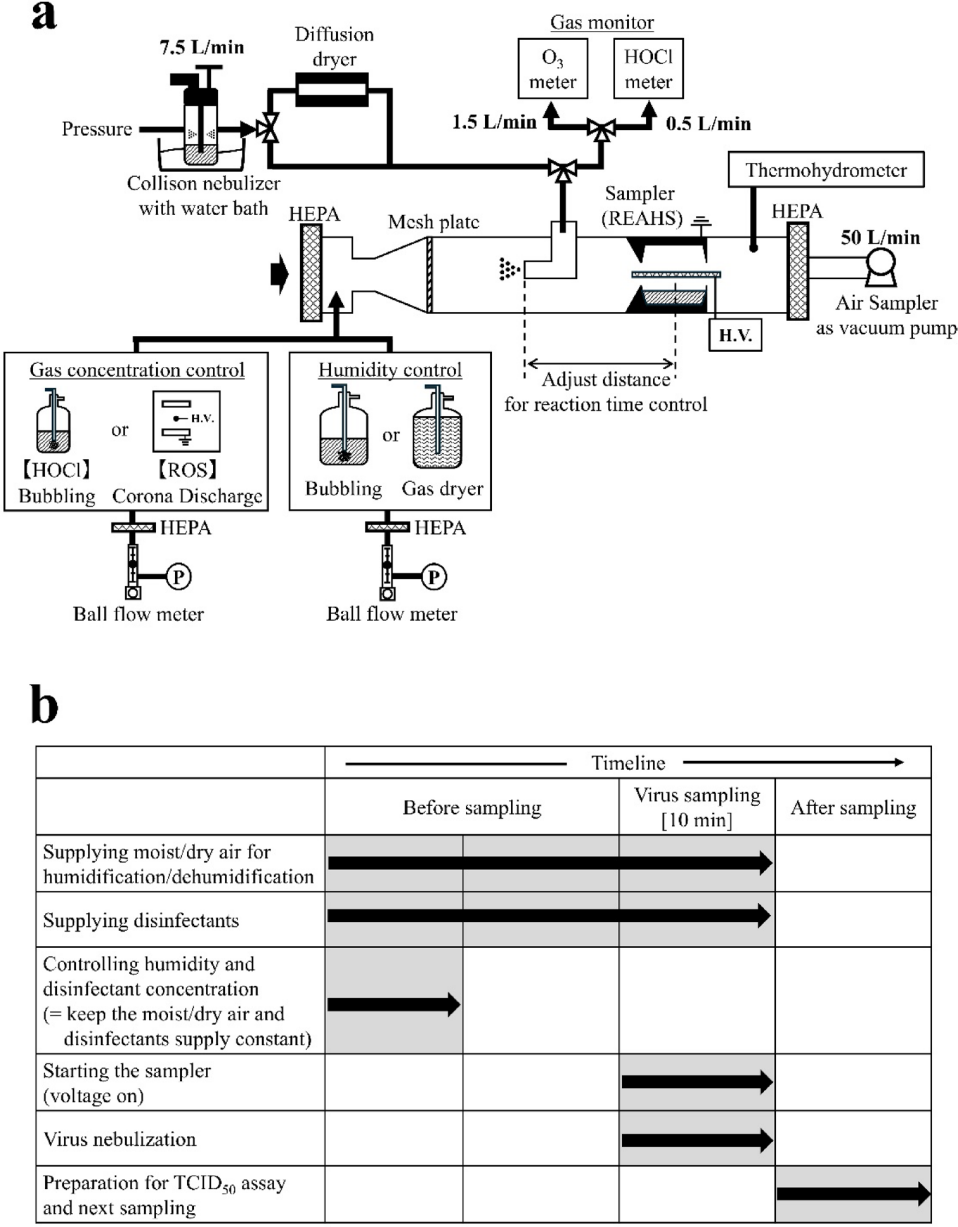
Experimental design for the inactivation effect. (**a**) Experimental set-up shows the system used to evaluate the inactivation effect. (**b**) Experimental timeline shows the sequence of operations for the inactivation experiment. The system was operated according to this timeline.

A duct system with a one-pass route was designed for evaluating the inactivation of airborne viruses (Fig. 4a). Air was drawn in at a flow rate of 50 L/min using a suction pump located at the downstream end, while purified air was supplied through a HEPA filter installed at the upstream end.

An environmental control unit positioned after the HEPA filter provided the duct with either humidified or dry air, along with gases for humidity control. Humidified air was generated by bubbling ultrapure water with clean air at a maximum flow rate of 32 L/min, while dry air was supplied at a maximum flow rate of 17 L/min by passing air through a glass bottle filled with silica gel. The flow rate was adjusted to achieve the desired humidity level using a temperature and humidity system within the duct. The temperature was maintained at approximately 24.8 ± 1.9 °C, which is the ambient temperature of the laboratory. The virus was introduced after these flow rate adjustments were made and stability was confirmed at an accuracy of ± 0.5% relative humidity. Humidity control was conducted in parallel with the gas concentration adjustments described below.

#### Disinfectant concentration control

The adjusted HOCl_(aq)_ was bubbled with clean air to produce volatilised HOCl_(g)_, which was then supplied to the duct. The concentration of HOCl_(g)_ in the air within the duct was measured at the virus supply point—where the virus was introduced into the duct—using a chlorine gas concentration meter (XPS-7; New Cosmos Electric Co., Ltd., Osaka, Japan)^39^. The flow rate of clean air supplied to the bubbling bottle was adjusted between 2 and 10 L/min to achieve the desired gas concentration based on readings from the chlorine gas concentration metre.

ROS were generated by supplying clean air at a flow rate of 5.0–8.5 L/min into the corona discharge box, with the air containing the generated ROS then introduced into the duct. At the virus supply point, the O₃ concentration was measured as a representative concentration of ROS using an O₃ concentration meter (measurement accuracy: 0.001 ppm; MODEL1200; Dylec Corporation, Ibaraki, Japan). The flow rate of the supplied clean air and the voltage applied to the discharge electrode were adjusted to achieve the desired O₃ concentration.

#### Aerosolisation

The virus was aerosolised using a collision nebuliser (CN25; CH Technologies, Westwood, NJ, USA). Briefly, 40 mL of the virus solution, adjusted to the desired concentration, was loaded into the nebuliser, which was pressurised at 0.05 MPa, and the solution was sprayed into the duct space via the virus supply unit. The nebuliser was placed in ultrapure water heated to 36 °C, and the solution was sprayed in this heated state to simulate human droplets. The particle size distribution of the aerosol was measured at 3 cm from the outlet of the L-shaped tube using a laser diffraction particle size analyser (Spraytech, STP2000; Malvern Panalytical, Malvern, UK). The volume mode diameter was 3.98 µm (Supplementary Fig. 1). Furthermore, the sprayed aerosol containing the H1N1 influenza A virus was passed through a diffusion dryer (DDU570; Topas GmbH, Dresden, Germany) before being introduced into the duct as dry aerosol.

#### Sampling

For virus sampling, a rotating wet electrostatic sampler developed by the authors and modified to accommodate the 50 L/min flow rate of the test system was used^47^. Compared to other methods, the electrostatic collection method offers advantages, such as low pressure loss and minimal stress on virus particles, resulting in less damage to the virus^48^. The sampler is supplied with liquid to recover the virus, which is collected into the liquid (Supplementary Fig. 2). Virus sampling was initiated simultaneously with virus spraying and continued for 10 min (Fig. 4b). After this period, liquid containing the virus was extracted from the sampler and evaluated using the TCID_50_ method. The sampler was operated at an applied voltage of 12 kV and rotation speed of 20 rpm. The liquid used for the HOCl test was 0.03% Na₂S₂O₃, and the ROS gas supply was 4 mL of 1 × PBS containing 0.3% Na₂S₂O₃. This ensured that the reaction between the virus and the gas was arrested. After 10 min without virus supply, the collected liquid showed no trace of available chlorine and nitrite ions did not increase.

As the flow rate was maintained at 50 L/min, the contact time with the gas was controlled by adjusting the distance between the sampler and the virus supply point. The flow rate in the duct was calculated accordingly, and tests were conducted under two conditions: approximately 1.8 s and 3.9 s, based on the distance between the centre of the sampler and the spray nozzle. These time settings were selected to correspond with the 1.9 s it takes for a sneeze to reach 1.1 m and the 4.18 s it takes for a cough to reach 1.5 m, as calculated using an approximate formula derived from previous research on the visualisation of sneezing and coughing^49^. The number of trials (n) for each condition is indicated in the figures or their captions. One trial comprises a paired set of measurements: one with the gas supplied at the specified concentration and one under the blank condition without gas supply.

### Calculation of the inactivation effect

The inactivation effect under each condition was determined by sampling under the same environmental conditions at a gas concentration of 0 ppb, which was taken as C_blank_. In the case of HOCl, ultrapure water was bubbled, and in the case of ROS gas, the voltage applied to the discharge electrode was set to 0 kV. C_blank_ sampling was performed for each condition and compared, and the SP and Log reduction were calculated according to the following formulae:

1 $$Survival\;Percent\;\left( {SP} \right)\;\left[ \% \right] = \frac{{C_{gas} }}{{C_{blank} }} \times 100$$

2 $$Log\;reduction\;\left[ - \right] = \log_{10} \frac{{C_{gas} }}{{C_{blank} }}$$

where C_gas_ is the infectious virus concentration at each gas load, and C_blank_ is the blank condition at a gas concentration of 0 ppb. The SP under each blank condition was set to 100 [%].

### Calculations and statistical analyses

Data are presented as mean ± standard deviation. Data were analysed using Origin Pro 2015 software (OriginLab, Northampton, MA, USA). The Wilcoxon signed-rank test was used to test for significant differences in the gas-induced inactivation effects. *p*-values < 0.05 were considered statistically significant, and all tests were one-tailed. Tests were performed by normalising the SP of each C_blank_ to 100%. The number of trials (*n*) and the *p*-value for each test result are indicated in the figure. In the boxplot, the box represents the 25^th^ to 75^th^ percentile range, the whisker plot shows the maximum and minimum values, the thick line within the box denotes the median, and the ■ symbol represents the average value. The number of trials was not uniform across all experimental conditions for the following reasons. First, we aimed to secure at least three trials within a limited number of attempts. Second, the number of trials for some conditions was increased during the course of the study, either as a result of comparing them with other conditions or to ensure reliability. It is important to consider that experimental conditions with fewer repetitions may show greater variability in results when interpreting the findings.

## Supplementary Information

Below is the link to the electronic supplementary material.

Supplementary Material 1

## Data Availability

The data supporting the findings of this study are not openly available due to sensitivity reasons. However, they are available from the corresponding author upon reasonable request. Data are stored in controlled access data storage at Panasonic Ecology Systems Co., Ltd.
