# Supplementary material for: Rapid inactivation of aerosolised influenza virus using low-concentration gaseous hypochlorous acid
Source: Sci Rep. 2025 Sep 29;15:33610. doi: 10.1038/s41598-025-19020-8 (PMC12480540; doi:10.1038/s41598-025-19020-8)
Supplement: Supplementary file 1 — Supplementary Material 1 [file 41598_2025_19020_MOESM1_ESM.pdf]

Supplementary information for

Rapid inactivation of aerosolised influenza virus using low-concentration gaseous hypochlorous acid

Koki Narihata[a,b], Masaru Minamiguchi[a], Miki Hata[a], Mitsuhiko Ueda[a], Shinji Yoshida[a, *], and Yoshihiro Sakoda[c,d,e,f, *]

[a]R&D Center, Indoor Air Quality Business Division, Panasonic Ecology Systems Co., Ltd., Kasugai 486-8522, Japan

[b]Green Transformation Division, Panasonic Holdings Corporation, Moriguchi 570-8501, Japan

[c]Laboratory of Microbiology, Department of Disease Control, Faculty of Veterinary Medicine, Hokkaido University, Sapporo 060-0818, Japan

[d]One Health Research Center, Hokkaido University, Sapporo 060-0818, Japan

[e]International Collaboration Unit, International Institute for Zoonosis Control, Hokkaido University, Sapporo 001-0020, Japan

[f]Hokkaido University Institute for Vaccine Research and Development (HU-IVReD), Hokkaido University, Sapporo 001-0021, Japan

*Corresponding authors: sakoda@vetmed.hokudai.ac.jp (Y.S.), yoshida.shinji002@jp.panasonic.com (S.Y.)

This PDF file includes:

Supplementary Figures 1–2

References [1]

Information Classification: General

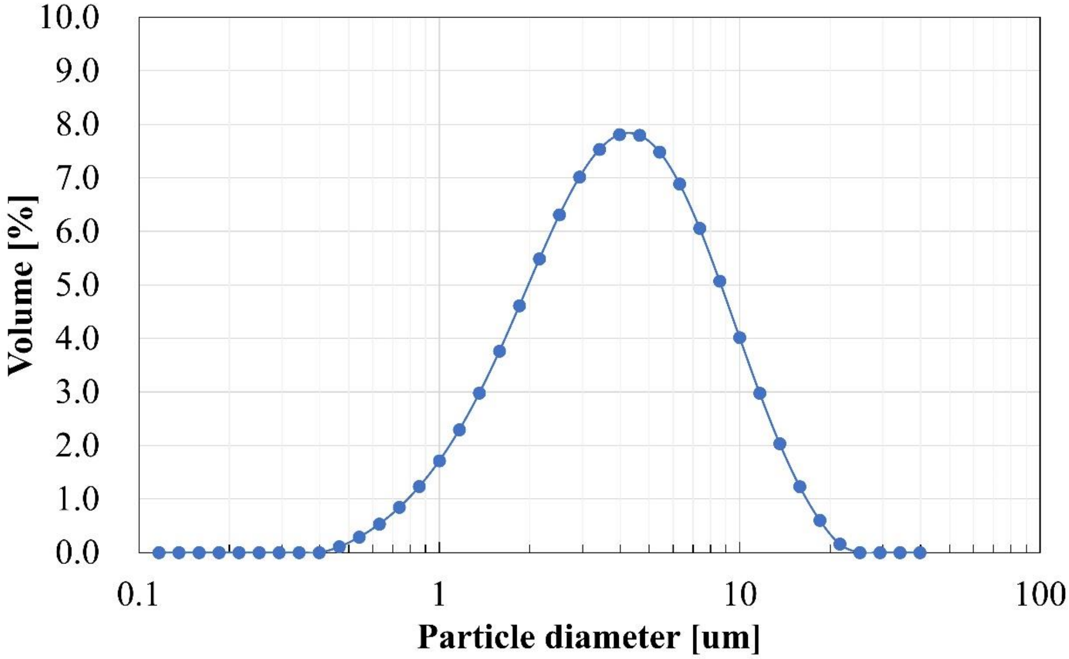

Figure S1. Volume particle size distribution.

Volumetric particle size distribution of the aerosol was measured at 3 cm from the outlet of the L-shaped tube using a laser diffraction particle size analyser (Spraytech, Malvern Panalytical, STP2000); these results are shown in Fig. S1.

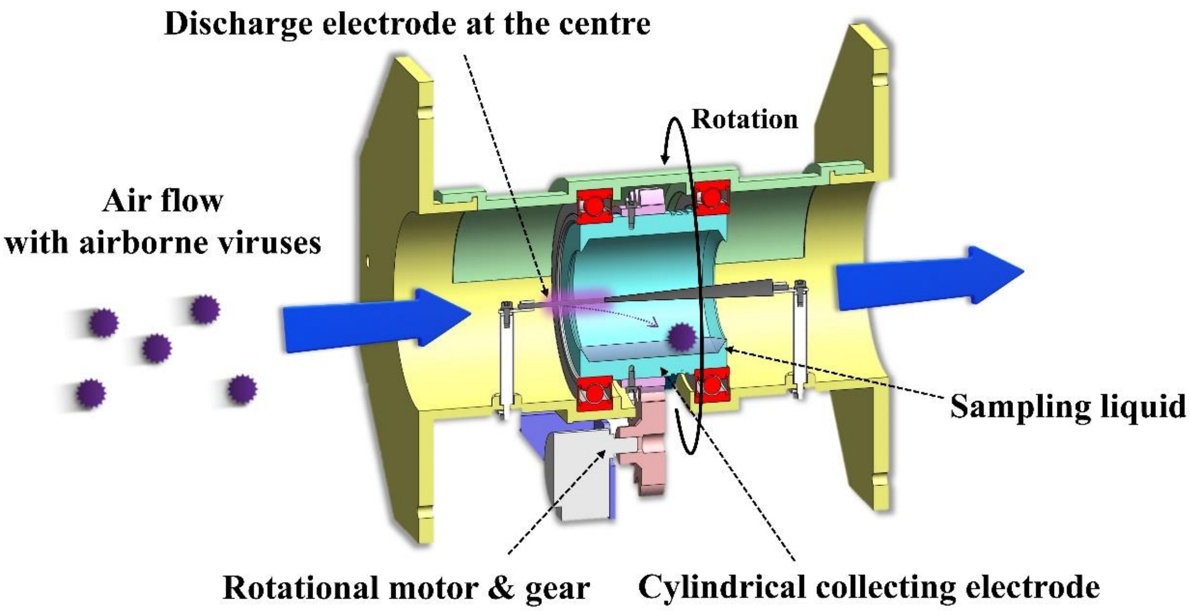

Figure S2. Cross-sectional view of the REAHS: Schematic of the sampling and collection into liquid

The detailed sampling process for the rotating electrostatic aerosol-to-hydrosol sampler (REAHS) is shown in Fig. S2 [1]. The REAHS uses a single-stage cylindrical electric collector with a combined electrode for both charging and collecting. A cylindrical collecting electrode was grounded, while a high voltage was applied to the discharge electrode at the centre, thereby forming an electric field between the two electrodes to generate a corona discharge. The incoming airborne virus particles were electrostatically charged by the positive ions generated by corona discharge. The charged particles are transported to the inner surface of the rotating cylindrical collecting electrode. This is a batch liquid-based sampler, with a fixed volume of sampling liquid supplied to the chamber. The virus on the electrode surface was transferred to the collecting liquid by rotating the electrode.

Reference

[1]. Narihata, K. & Iida, K. Development of electrostatic aerosol-to-hydrosol sampler for biosensing of airborne influenza virus. *Earozoru Kenkyu* **36**, 253–262 (2021).

Information Classification: General
